# Editorial: Localization and scene understanding in urban environments

**DOI:** 10.3389/frobt.2024.1509637

**Published:** 2024-11-08

**Authors:** Augusto Luis Ballardini, Daniele Cattaneo, Domenico G. Sorrenti, Ignacio Parra Alonso

**Affiliations:** ^1^ Computer Engineering Department, Universidad de Alcalá, Alcalá de Henares, Spain; ^2^ Department of Computer Science, University of Freiburg, Freiburg, Germany; ^3^ Department of Informatics, Systems and Communication DISCO, Università degli Studi di Milano - Bicocca, Milan, Italy

**Keywords:** GNSS-denied environments, openstreetmap, localization, HD-map, traffic light, extended Kalman filter, CUDA, self-driving

Urban settings represent one of the main challenges for self-driving vehicles today. Navigating a vehicle in these environments with only Global Navigation and Satellite Systems (GNSS) can lead to potentially dangerous situations that could jeopardize the safety of both onboard passengers and road users. All navigation systems rely on two main aspects: a reference map and a localization system, which uses the latter for identifying the position of the vehicle.

Regarding the reference map, nowadays different proposals from mapping companies such as TomTom^1^ and Here^2^, as well as self-driving companies like Waymo^3^, are paving the way for reliable sources of information. On the other hand, open-source community-created geospatial projects like OpenStreetMap are also a valuable source of information that have been extensively used for research purposes over the last decade. Localization algorithms are responsible for accurate localization of the vehicle within the map. This is achieved using different on-board sensors, starting with the most obvious: GNSS. Despite these systems’ ability to provide very good localization accuracies, up to the order of 0.01 m, their reliability Research Topic place them in the danger tier, especially in urban areas. This is due to the need for a clear line of sight to a significant part of the sky, which can be often obstructed by trees, buildings or urban canyons or even thick clouds.

Matteo Frosi et al. investigated this aspect in their first contribution, focusing on the evaluation of the positioning accuracy and precision of localization systems that are not based on GNSS sensors. Besides presenting also a novel Simultaneous Localization and Mapping (SLAM) system that achieves real-time performance, the authors benchmarked a set of algorithms on manually collected datasets, demonstrating the active interest of the scientific community in the Research Topic. They presented a comparison of algorithms that use LiDAR data coupled with inertial measurements units, a technique that has been extensively investigated over the last decade. Results from both outdoor and indoor scenarios show very promising results for real-world applications such as autonomous driving.

In a second contribution, a different group also led by Frosi et al., proposed an algorithm that directly connects with the HD mapping concept previously described. Among all the features that HD maps integrate, they interestingly used one of the major sources of problems: the buildings themselves. They extended a SLAM system to exploit mapped buildings in OpenStreetMap by matching single LiDAR scan inputs. Their contribution also presented the re-localization capabilities of their systems. This approach is interesting and paves the way to further investigation in the field, as similar techniques could also be used for other elements of mapped environments.

In this perspective, the work of the group led by Mentasti et al. proposes a pipeline to enrich HD-maps with traffic lights, enhancing their detection with specific features like shape, orientation, and pictogram. Unlike the previous two contributions, their focus is leveraging image information from onboard cameras on surveying vehicles and state-of-the-art deep neural networks for traffic light detection. Furthermore, to accommodate GNSS errors during the mapping process, the authors proposed to use Kalman filtering techniques to provide the most accurate traffic light position possible.

Even though localization is, in a way, a lower scale of complexity compared to SLAM algorithms, as they exploit already available maps, all the algorithms presented so far use special techniques that require high computational resources. Most of the time, parts of these algorithms include high-consuming subprocesses due to repetitive massive sets of operations performed on the sensed data. This can be the case of either LiDAR input with thousands of 3D points as well as imagery data, processed using deep neural networks. While specific hardware is available to speedup inference and classification of images, the same hardware can be used to parallelize consolidated filtering techniques. This is demonstrated in the last work presented in this collection, where Mochurad proposed an implementation of a parallel Kalman filter algorithm to speedup vehicle localization based on a specific usage of the LiDAR data. Using the CUDA programming language and NVIDIA hardware, her proposal achieved a 3.8x speedup compared to the original un-parallelized implementation of the Kalman algorithm, which is remarkable as it allows easy scaling with the number of 3D LiDAR points used for localization.

This Research Topic sought to explore the vehicle localization field from a variety of perspectives, stimulating the scientific community to exploit different cues from common urban settings. We foresee that the future of self-driving will strongly rely on localization algorithms that exploit a comprehensive understanding of the surrounding environment. Only through global awareness will it be possible to preemptively identify dangerous conditions that might lead to non-fatal injuries or deaths. We hope that this short collection can inspire the readers and researchers to push the boundaries in the realm of vehicle localization.

## Footnotes

1
https://www.tomtom.com/products/hd-map (Accessed October 29, 2024)

2
https://www.here.com/platform/HD-live-map (Accessed October 29, 2024)

3
https://waymo.com/blog/2016/12/building-maps-for-self-driving-car (Accessed October 29, 2024)

